# The Primitive Thylakoid-Less Cyanobacterium *Gloeobacter* Is a Common Rock-Dwelling Organism

**DOI:** 10.1371/journal.pone.0066323

**Published:** 2013-06-18

**Authors:** Jan Mareš, Pavel Hrouzek, Radek Kaňa, Stefano Ventura, Otakar Strunecký, Jiří Komárek

**Affiliations:** 1 Institute of Botany ASCR, Centre for Phycology, Třeboň, Czech Republic; 2 Department of Botany, Faculty of Science, University of South Bohemia, České Budějovice, Czech Republic; 3 Institute of Microbiology ASCR, Department of Autotrophic Microorganisms - ALGATECH, Třeboň, Czech Republic; 4 CNR-ISE Istituto per lo Studio degli Ecosistemi, Sesto Fiorentino, Italy; 5 Centre for Polar Ecology, Faculty of Science, University of South Bohemia, České Budějovice, Czech Republic; Louisiana State University and A & M College, United States of America

## Abstract

Cyanobacteria are an ancient group of photosynthetic prokaryotes, which are significant in biogeochemical cycles. The most primitive among living cyanobacteria, *Gloeobacter violaceus*, shows a unique ancestral cell organization with a complete absence of inner membranes (thylakoids) and an uncommon structure of the photosynthetic apparatus. Numerous phylogenetic papers proved its basal position among all of the organisms and organelles capable of plant-like photosynthesis (i.e., cyanobacteria, chloroplasts of algae and plants). Hence, *G. violaceus* has become one of the key species in evolutionary study of photosynthetic life. It also numbers among the most widely used organisms in experimental photosynthesis research. Except for a few related culture isolates, there has been little data on the actual biology of *Gloeobacter*, being relegated to an “evolutionary curiosity” with an enigmatic identity. Here we show that members of the genus *Gloeobacter* probably are common rock-dwelling cyanobacteria. On the basis of morphological, ultrastructural, pigment, and phylogenetic comparisons of available *Gloeobacter* strains, as well as on the basis of three new independent isolates and historical type specimen, we have produced strong evidence as to the close relationship of *Gloeobacter* to a long known rock-dwelling cyanobacterial morphospecies *Aphanothece caldariorum*. Our results bring new clues to solving the 40 year old puzzle of the true biological identity of *Gloeobacter violaceus*, a model organism with a high value in several biological disciplines. A probable broader distribution of *Gloeobacter* in common wet-rock habitats worldwide is suggested by our data, and its ecological meaning is discussed taking into consideration the background of cyanobacterial evolution. We provide observations of previously unknown genetic variability and phenotypic plasticity, which we expect to be utilized by experimental and evolutionary researchers worldwide.

## Introduction

Cyanobacteria are the most significant group of photosynthetic prokaryotes, generating global impact effecting biogeochemical cycles since ancient Earth history [1], [2]. As one of the most important sources of atmospheric oxygen and crucial carbon fixers, they have been intensively studied by experimental and evolutionary science [3], [4], [5]. *Gloeobacter violaceus* Rippka et al. 1974, the most primitive among living cyanobacteria, has been subjected to both of these approaches. In its original description [6], the authors studied a single cyanobacterial strain (PCC 7421) isolated from the surface of a limestone rock in Kernwald (Switzerland). A peculiar simple cell organization with complete absence of thylakoids and an unusual structure of the photosynthetic apparatus supported the description of a new, separate monotypic genus *Gloeobacter* [6], [7], [8]. Following studies of the phylogenetic comparison of SSU rRNA gene and other loci [9], [10], [11] demonstrated that *G. violaceus* diverged very early during the cyanobacterial radiation, in an ancient lineage preceding the cyanobacterial chloroplast ancestors [12]. These findings were in accordance with its primitive morphology and cell ultrastructure. Since then, *G. violaceus* PCC 7421 has become one of the key species in evolutionary studies of (cyano)bacteria [13], [14], [15] and plant life in general [16], [17], [18]. *Gloeobacter* was also among the first cyanobacterial strains having its complete genome sequenced [19].

Besides its significance in evolutionary research, *G. violaceus* PCC 7421 has been frequently used as a model organism for experimental studies of oxygenic photosynthesis [20]. A unique molecular structure of photosystems I and II [21], [22], [23] and an unusual morphology of its phycobilisomes (PBS) [24] enable *Gloeobacter* to harvest light and transfer energy in a manner, which is different from other photosynthetic organisms. Unlike in other cyanobacteria, its PBSs are composed of six peripheral phycocyanin/phycoerythrin rods bound as a bundle to five horizontal rods of an allophycocyanin core, allowing atypical energy transfer pathways [25]. Apart from the field of photosynthesis research, a pentameric ligand-gated ion channel (GLIC) was cloned from *G. violaceus* and has become an important molecular model of membrane receptors in general biological and clinical studies [26], [27], [28].

In contrast to detailed knowledge of cell structure, physiology and genetics of the experimental model strain PCC 7421, data on the ecology, distribution, life strategy and overall variability in the genus *Gloeobacter* have, until now, remained extremely limited. Apart from PCC 7421, a few more populations were collected and isolated from nearby localities (PCC 9601, PCC 8105). The only cyanobacterium morphologically and phylogenetically corresponding to *G. violaceus* found, aside from the original locality, was reported to be from a surface of a fountain in Florence, Italy [29]. The unexplained biological identity of *Gloeobacter* and its relationship to other cyanobacteria has been under discussion ever since the establishment of the genus. Already in its original description, Rippka et al. [6] suggested a close relationship to a little known botanical species *Gloeothece coerulea* Geitler 1927. This latter cyanobacterium has a similar, simple cell morphology and life cycle, and contains polar granules clearly resembling those of *G. violaceus* [30]. On the basis of this assumption and the observation of fossil cyanobacterial remnants, Golubic & Campbell [31] hypothesized that the *Gloeobacter*/*Gloeothece coerulea*-like morphotype has colonized epilithic habitats since the Precambrian. Another rock-inhabiting species, *Aphanothece caldariorum* Richter 1880, was studied in detail by Hansgirg [32] and it has quite often been found in samples of (sub-)aerophytic microalgal biofilms since then. As noted by Komárek & Anagnostidis [33], this cyanobacterium is almost identical to *G. coerulea* in both morphology and life strategy and thus potentially related to *G. violaceus*.

Under the current cyanobacterial taxonomy [33], the genus *Gloeobacter* can be relatively easily distinguished from the morphologically similar genera *Aphanothece, Anathece, Cyanobium* and *Gloeothece* by its phylogenetic position and by the absence of thylakoids. However, until now, *Gloeobacter* was studied exclusively by observing its cultures, while similar morphospecies from other genera (e.g., *Gloeothece coerulea*, *Aphanothece caldariorum*) have never been isolated into cultures, making it impossible to study them using electron microscopy and molecular analysis to provide the necessary evidence concerning their phylogeny and cell ultrastructure.

In this study, we provide extensive evidence for three original isolates of *Gloeobacter violaceus*, a botanical type specimen of *A. caldariorum*, and two reference strains *G. violaceus* PCC 7421 and PCC 9601 to uncover their taxonomic identity, phylogeny, and biology in natural populations.

## Methods

### Strains and Samples

Two strains of *Gloeobacter violaceus* (PCC 7421 and PCC 6901) were obtained from the Pasteur Culture Collection of Cyanobacteria, Paris, France. Another strain (VP3-01) was purified from a biofilm growing on a surface of a fountain in Florence, Italy, as previously described [29]. Two new strains were isolated from samples dominated by cyanobacteria corresponding to the morphospecies *Aphanothece caldariorum* from wet rocks in artificial waterfalls in tropical greenhouses of botanical gardens in Liberec and Teplice, the Czech Republic, in the years 2009–2010. The collection of samples of cyanobacteria in the greenhouses of the public Botanical Gardens in Liberec and Teplice, the Czech Republic was approved by their directors Miloslav Studnička (Liberec) and Jiří R. Haager (Teplice). No additional permissions were required by the legal regulations of the Czech Republic. No protected species were sampled. For isolation, a portion of an environmental sample was spread on the surface of an agar plate enriched with BG 11 medium [34]. Colonies that emerged from individual *Aphanothece caldariorum*-like cells (checked by optical microscopy) were then sequentially transferred to fresh plates until unicyanobacterial strains were obtained. The holotype of *A. caldariorum* var. *cavernarum* Hansgirg 1889 was provided by the WU (Institute of Botany, University of Vienna) herbarium. The type material of the nominate variety of this species was not found in the respective herbaria, as it was probably lost.

The strains were cultivated in both liquid and agar BG11 medium for morphological, ultrastructural and molecular analysis. For the purpose of pigment analysis and statistical analysis of cell dimensions, the strains were grown in 50 mL liquid batch cultures (inoculated with 1 mL of a previous batch at growth maximum), under constant temperature (23°C) and irradiance (20±5 µmol m^−2^ s^−1^). Our original cultures were deposited in Culture Collection of Autotrophic Organisms (CCALA) of the Institute of Botany ASCR, which is accessible to the public, under accession codes CCALA 979 ( = *G. violaceus* VP3-01 from Florence), CCALA 980 ( = *G. violaceus* [*A. caldariorum* morphotype] from Teplice) and CCALA 981 ( = *G. violaceus* [*A. caldariorum* morphotype] from Liberec).

### Morphology and Ultrastructure

Fresh cyanobacteria were observed under 400× and 1000× magnifications using Olympus BX 51 microscope equipped with differential interference contrast, an Olympus DP71 camera, and the QuickPhoto Micro v. 2.3 image analysis software. Statistical analysis of cell dimensions in cultures was based on photographs taken at 1000× magnification. Length and width of 100 randomly selected mature cells (excluding the stage of fission or just after it) from each sample were measured with an accuracy of ±0.1 µm. Statistical differences in cell length and length:width ratio between the batch cultures of individual strains was assessed, using Statistica v. 9.1 [35], by a Kruskal-Wallis test; pair-wise differences among the batches were evaluated by standard 2-tailed t-tests.

For ultrastructural studies, biological material of cyanobacteria was fixed with 6% glutaraldehyde and kept at room temperature. Samples were washed with 0.05 M phosphate buffer (pH 7.2) and postfixed with 2% osmium tetroxide in the same buffer at room temperature for 2 hours, then repeatedly washed with 0.05 M phosphate buffer. Finally, cells were dehydrated with a graded isopropanol series and embedded in Spurr’s resin [36] using propylene oxide as an intermediate stage. Thin sections were stained with uranyl acetate and lead citrate and observed in a Jeol JEN 1010 transmission electron microscope at 80 kV.

### Molecular Analysis

The biomass was dried for 48 hours over silica gel and crushed to powder in a Retsch MM200 laboratory mill with wolfram carbide beads (3 minutes, 30·s^−1^). Total genomic DNA was isolated following the modified xanthogenate-SDS buffer extraction protocol with addition of 3% PVPP and PEG-MgCl_2_ precipitation [37]. Alternatively, for the historical type specimen of *A. caldariorum* var. *cavernarum*, a small piece (1 mm^2^) of the herbarium material was pulverized as previously, rehydrated in 50 µL of TE buffer, and 2 µL of the suspension were directly added to the PCR mix. A section of the rRNA operon containing the partial SSU rRNA gene and the ITS region was amplified with primers 359F and 23S30R [38], [39] (Table 1). Ten ng of template DNA was mixed with 6 pmol of each primer in a commercial PCR mix with Taq polymerase (Plain PP Master Mix, Top Bio, the Czech Republic), and amplified with an initial denaturation step (5 minutes at 95°C), 35 cycles of denaturation (1 min at 94°C), primer annealing (45 s at 55°C) and elongation (2 min at 72°C), and final elongation for 10 min at 72°C. The PCR product was cloned using the standard pGEM-T Easy (Promega Corp., WI, USA) vector system according to supplier instructions. The plasmid containing the required insert was purified from the bacterial culture using Zyppy Plasmid Miniprep kit (Zymo Research Corp., CA, USA). The *rpo*C1 gene fragment was amplified using primers rpc/MF and rpc/CR-1 (Table 1) following the published protocol [9] (initial denaturation for 5 min at 94°C, 35 cycles of 1 min denaturation at 94°C, primer annealing for 1 min at 52°C, elongation for 2 min at 72°C, and final elongation step for 10 min at 72°C), and cloned as above. PCR gel pictures are given in Figure S4. The clones were sequenced using primers T7 and SP6r (Table 1) in Laboratory of Genomics, Biology Centre of the Academy of Sciences of the Czech Republic, České Budějovice (with an ABI PRISM 3130 XL, Applied Biosystems, Life Technologies Corp., CA, USA). Sequences were deposited in GenBank under accession numbers KC004017–KC004023 and KC866356.

**Table 1:** List of PCR and sequencing primers.

Primer Name	Sequence (5′to 3′)
**359F** 1 [39]	GGG GAA TYT TCC GCA ATG GG
**23S30R** 1 [38]	CTT CGC CTC TGT GTG CCT AGG T
**Cyano6r** ^2^ *	GAC GGG CCG GTG TGT ACA
**T7** 2	TAA TAC GAC TCA CTA TAG GG
**SP6r** 2	TAT TTA GGT GAC ACT ATA G
**rpc/MF** 1 [9]	GGT GAR GTN ACN AAR CCA GAR AC
**rpc/CR-1** 1 [9]	CCA GAR TAG TCN ACC CGT TTA CC

1 PCR primers,

2 sequencing primers,

* reverse complement to primer 14 [38].

### Phylogenetic Analysis

Sequences of the SSU rRNA and *rpo*C1 gene from thirty-eight cyanobacteria were collected from published studies and mined from the whole genome database available in GenBank. The sequence matrices were assembled to include equal number of strains from all cyanobacterial orders. Three bacterial strains, *E. coli* K12, *Salmonella enterica* str. UK-1, and *Cronobacter turicensis* z3032, were used as out-group taxa. The sequences were aligned via MAFFT v. 6 [40] using the FFT-NS-I strategy, and manually corrected. For the final analysis of the SSU rRNA gene data, a region of alignment that is common to all collected sequences was used. It spanned 1041 positions from 377 to 1432 (*Escherichia coli* numbering) after ambiguous gap columns were removed. Phylogenetic analysis was conducted employing Bayesian inference in MrBayes 3.1.2 [41], maximum likelihood analysis in RAxML 7.3.2 [42], and maximum parsimony analysis in PAUP* 4.0b10 [43]. For the Bayesian analysis, two runs of four Markov chains were executed for 1 000 000 generations with default parameters, sampling every 100 generations (the final average standard deviation of split frequencies was lower than 0.01). The maximum likelihood calculation was executed upon the generalized time-reversible (GTR) substitution model with discrete gamma distribution in six categories. The gamma shape parameter α as well as the proportion of invariable sites were estimated from the data set (GTR+Г+ G model), and 1000 bootstrap replicates were calculated to evaluate the relative support of branches. A maximum parsimony analysis involved one hundred replicate searches with starting trees obtained by random stepwise addition, using the tree bisection-reconnection (TBR) branch swapping algorithm; one thousand nonparametric bootstrap replications were run with the same settings to evaluate the relative branch support. All bases and base changes were equally weighted, and gaps were coded as missing data. MetaCentrum (www.metacentrum.cz) and CIPRES (www.phylo.org) supercomputing facilities were used for fast calculation of Bayesian and likelihood trees.

For the combined analysis of both loci, a 778 bp long region of partial *rpo*C1 data was merged with an aligned SSU rRNA gene from corresponding strains into a final concatenated alignment of 1988 bp. Phylogenetic analysis of the concatenated alignment was conducted using Bayesian inference, maximum likelihood and maximum parsimony methods as mentioned previously. Phylogenetic trees were visualized using FigTree v. 1.3. (http://tree.bio.ed.ac.uk/software/figtree/). The alignments were uploaded to the TreeBase web (http://purl.org/phylo/treebase/phylows/study/TB2:S14106 ).

### Pigment Analysis

The relative concentrations of the two phycobiliproteins, phycocyanin (PC) and allophycocyanin (APC) were estimated from the mean absorption of light between 616–624 nm and 650–658 nm respectively according to Krogmann et al. [24]. The relative concentration of phycoerythrin (PE) was calculated at 662 nm, by utilizing an equation, which was used in the same study, as [PE] = A_662_/ε_662_ with ε_662_ (extinction coefficient) at 456 mM cm^−1^. Absorption spectra were recorded *in vivo* with a Unicam UV 550 (Thermospectronic, UK) following the method described by [44].

Fluorescence emission spectra of living cells at room temperature were measured using an Aminco-Bowman Series 2 spectrofluorometer, in the standard instrument geometry with excitation at 492 nm. Fluorescence emission was scanned between 550–750 nm with a 4 nm bandwidth.

To assess carotenoid composition, the biomasses of CCALA 981 and PCC 7421 were extracted with an acetone/methanol mixture (7/3 v/v) in the dark into Eppendorf tubes and centrifuged. The samples were subjected to HPLC analysis on the Agilent Technologies 1200 series chromatographic system with a diode-array detector. Separation was performed on a Luna C8 column (3 µm, 100A, 100×4.6 mm –00D-4248-E0, Phenomenex, USA) using methanol (A) and 28 mM amonium acetate in 80% methanol (B) as solvents (from 30% to 100% of solvent A in 30 min.). The pigments were detected on the basis of their retention times (internal database) and absorption spectra.

## Results and Discussion

### Natural Morphology and Habitat

**Figure 1:**
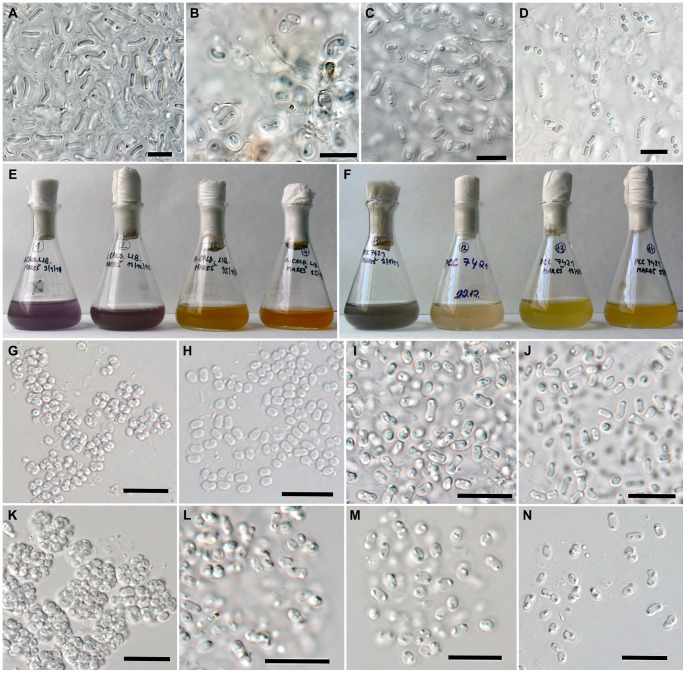
Morphology of *Gloeobacter violaceus* and *Aphanothece caldariorum*-like samples. (A) and (B) *A. caldariorum*-like samples from the botanical gardens in Liberec and Teplice, respectively, showing typical rod-shaped cells with polar granules and layered mucilaginous envelopes; (C) cell morhology in the perfectly preserved herbarium type specimen of *A. caldariorum* var. *cavernarum*; (D) “nanocytes“ in the environmental sample from Liberec dominated by *A. caldariorum*-like morphotype; (E) and (F) batch cultures ordered by increasing age (from the left) of *A. caldariorum*-like strain CCALA 981 and *G. violaceus* PCC 7421, respectively, showing a gradual color shift from grey-violet to yellow-orange; (G–J) and (K–N) change in cell morphology from subspherical nanocyte-like cells to rod-shaped cells with occasional mucilaginous envelopes in the batch cultures of CCALA 981 and PCC 7421, respectively (the batches are the same as in panels E and F). Scale bars, 10 µm.

Material collected from artificial waterfalls in greenhouses of two botanical gardens revealed the presence of a morphotype exactly matching the species *Aphanothece caldariorum* as described in botanical literature [32], [33]. In both cases, it was a component of an epilithic gelatinous cyanobacterial mass. In the native state, the collected material (from both greenshouses) showed all of the morphological characteristics typically described for *A. caldariorum* [33], such as dimensions and shape of cells, concentrically lamellated mucilaginous envelopes, and polar granules (Figure 1A, B). Compared to the typical properties of *Gloeobacter violaceus* strains [6], [33], the *A. caldariorum*-like cells were significantly longer (up to more than 10 µm versus 2–3 µm in *G. violaceus*), often somewhat bent or arcuate (straightly rod-shaped in *G. violaceus*), with broader and more conspicuously lamellated sheaths. We also recorded frequent occurrence of small subspherical cells, (Figure 1D) clearly corresponding to “nanocytes“ [33], which were not reported for *G. violaceus*. According to our observations, these cells do not seem to represent any specialized reproductive stage. Apparently, they were produced by fast serial binary fission under favorable conditions. *A. caldariorum*-like morhotype was found on wet rocks in waterfalls, which corresponded well to the original description of this species from botanical gardens in Prague and wet rocks in Bohemia [32], and also to other reports of this species from numerous similar localities worldwide [45], [46], [47], [48], [49], [50], [51], [52], [53]. The similarity of the morphotype to *A. caldariorum* was further confirmed through microscopic examination of the well-preserved botanical type specimen of *A. caldariorum* var. *cavernarum* (Figure 1C). Unfortunately, the type specimen of the nominate variety *caldariorum* was lost or destroyed during the Second World War, and is not available for study. Thus, a definite proof of identity (by molecular methods) of our material to *A. caldariorum* cannot be given.

### Strain Morphology and Pigment Analysis

Very high similarity in basic morphometric characteristics was found between typical *Gloeobacter* PCC 7421 strain and all the studied *Apahnothece caldariorum* morphotype samples. The observed cyanobacterial isolates also exhibited almost identical changes in morphology and pigmentation during their life cycle, as described below.

In cultures grown in nutrient-rich media (BG 11), cells resembling *Aphanothece caldariorum,* immediately after inoculation, started accelerated proliferation (Figure S1), resulting in compact clusters of small subspherical “nanocyte-like“ cells, mostly with two distinct granules inside (Figure 1G). As long as it was cultured in fresh media, this morphotype was maintained. Upon consulting available literature [6], [34], this strain morphology obviously matched that of the reference strain *Gloeobacter violaceus* PCC 7421. Our optical microscopy observations verified identical appearance of all gathered strains (PCC 7421, PCC 9601, CCALA 979, CCALA 980, CCALA 981– Figure 1G–N, Figure S2).

During the growth of batch cultures on both solid and liquid media, a dramatic color shift was observed in all strains (Figure 1E, F). The color changed from various shades of grey, greyish-blue-green and greyish-violet in young cultures, through bright violet or pinkish-violet at growth maxima, to green or yellow-green in older cultures, ending with yellow and orange in their stage of senescence. These observations were in accordance with reports of an extreme color variability of *A. caldariorum* in natural habitats [32], [33].

**Figure 2:**
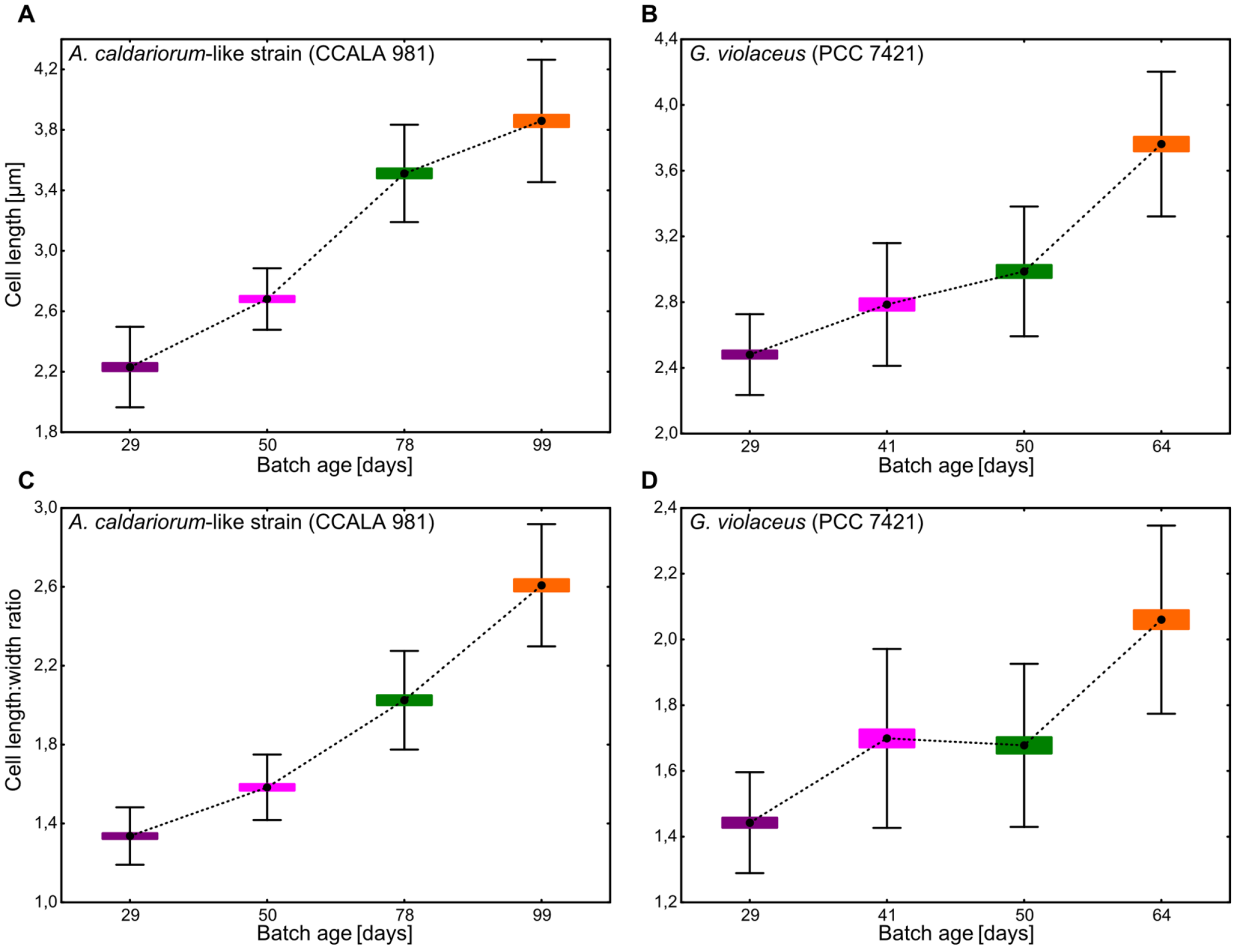
Morphometric analysis of cells in *Aphanothece caldariorum*-like and *Gloeobacter violaceus* cultures. (A) and (B) increasing cell length corresponding to batch cultures of increasing age of *A. caldariorum*-like strain CCALA 981 and *G. violaceus* PCC 7421 (Kruskal-Wallis test, p<0.001), difference between individual pairs of batches was also significant (two-tailed t-test, p<0.02 except for a pair of batches 41 and 50 days old in PCC 7421, for which the difference was at the edge of statistical significance, p = 0.045); (C) and (D) increasing cell length:width ratio in the same batch cultures as previously (Kruskal-Wallis test, p<0.001), difference between individual pairs of batches was also significant (two-tailed t-tests, p<0.001) except for a pair of batches 41 and 50 days old in PCC 7421. One hundred cells were measured in each sample.

In order to determine the principles and symptoms of these changes, we compared series of batch cultures grown in standardized conditions for one representative of typical *G. violaceus* (PCC 7421) and one representative of a strain derived from the *A. caldariorum*-like morphotype (CCALA 981). Four batches of different ages spanning the color range from young and thriving (grey, violet) to senescent cultures (yellow, orange) were chosen for morphometric and pigment analysis for each of the two strains (Figure 1E, F). As shown by the statistical analysis of cell dimensions (Figure 2, p<0.001), the cells of both strains changed their shape from subspherical to short cylindrical of “nanocytes“ in young cultures, to rod-shaped cells in old cultures (Figure 1G–N). Cultivated cells never reached a length of over 5 µm, which is a dimension frequently exceeded in natural populations of *A.caldariorum* (Figure 1A–C, [33]). A probable reason for such a difference lies in the unnatural chemical composition of the culture medium, and, possibly, also in the different physical properties of the artificial medium as compared to natural substrates. However, in the oldest batches the cells were often somewhat arcuate and enclosed in gelatinous envelopes (Figure 1J, N) clearly resembling the typical *A. caldariorum* morphotype.

To elucidate the process of color shift, we performed a light spectroscopy analysis of the main photosynthetic pigments (phycobilins and chlorophyll *a*) and HPLC analysis of carotenoids in the same batch cultures that were used for morphometry.

**Figure 3:**
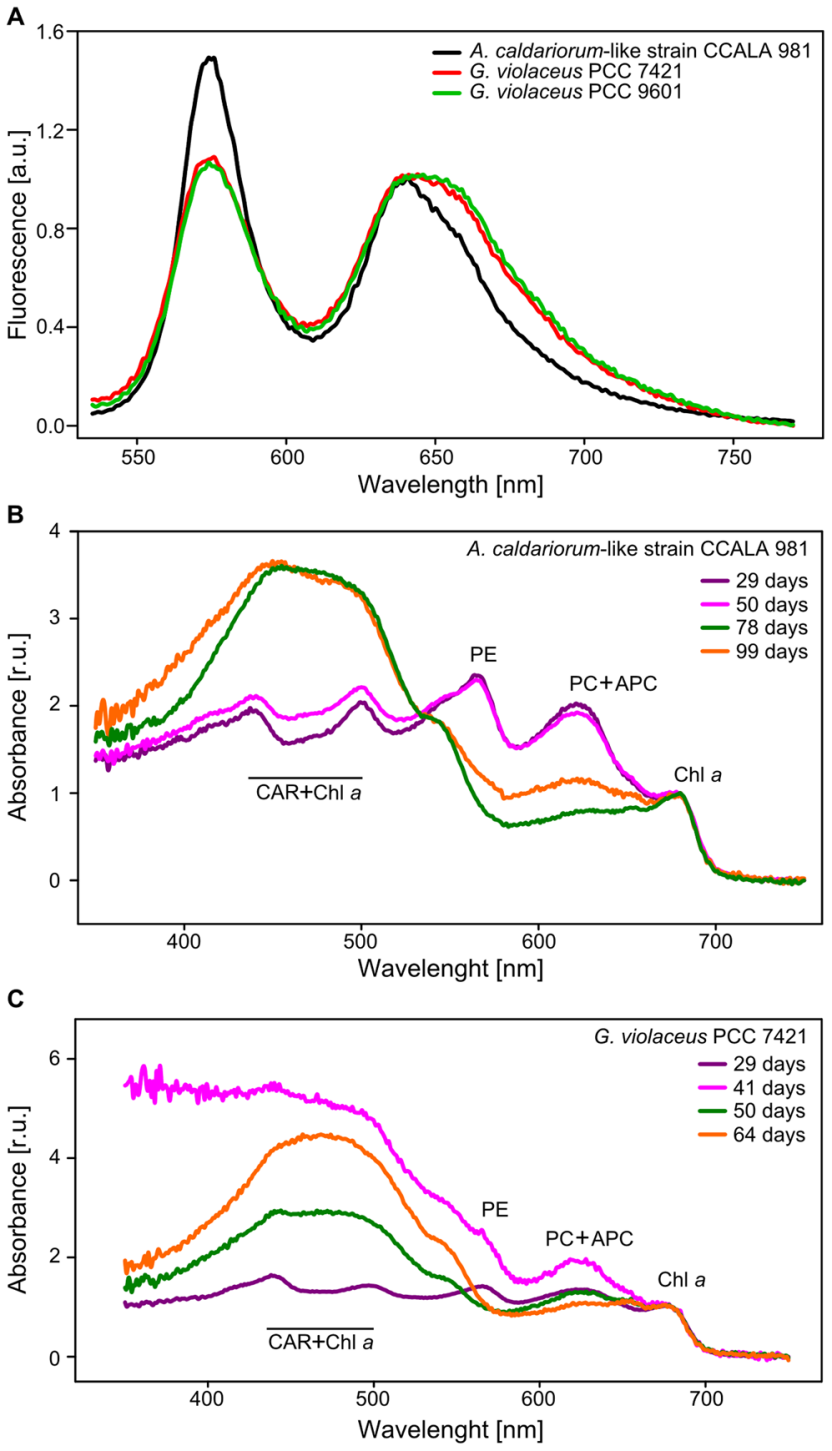
Light spectroscopy analysis of photosynthetic pigments in *Aphanothece caldariorum*-like strain and *Gloeobacter violaceus*. (A) fluorescence emission spectra of *A. caldariorum*-like strain CCALA 981 in comparison with *G. violaceus* PCC 7421 for excitation to phycobilisomes. Higher content of phycoerythrin in CCALA 981 is documented by a relative increase in its fluorescence emission at 574nm. (B) and (C) whole cell absorption spectra of CCALA 981 and PCC 7421 normalized to chlorophyll *a* content. Clear accumulation of carotenoids (wide absorbance peak between 450–500 nm) at the stationary phase of growth (third and fourth batch) is obvious in both strains. The absorbance of 41-day old strain PCC 7421 is raised due to the beginning of accumulation of carotenoids in this batch, but phycobilin peaks are still recognizable. Individual absorbance peaks are as noted; CAR, carotenoids; PE, phycoerythrin; PC+APC, phycocyanin and allophycocyanin; Chl *a*, chlorophyll *a*.

In general, the pigment composition of CCALA 981 and PCC 7421 was similar. Nevertheless, the *A. caldariorum*-like strain had slightly different molar ratios of particular phycobiliproteins within the PBS in comparison to *G. violaceus* PCC 7421 (Table 2). The molar ratio (measured in young cultures) in PCC 7421 was about 1APC: 1.3PC: 1.2 PE, while in CCALA 981 we found relatively higher contents of phycoerythrins and phycocyanins in comparison to allophycocyanins (1APC: 1.3 PC: 1.7 PE). The higher content of phycoerythrin in the latter strain was also confirmed by a relative increase in its fluorescence emission at 574nm (Figure 3A). The observed discrepancy in the phycobilin ratios corresponded well to a slightly different color of the cultures (violet-grey vs. bright violet). This finding suggests a relative increase in the length of PBS rods in CCALA 981 (Table 3). The PBS in *G. violaceus* PCC 7421 was previously shown to consist of 16 APCs in the core, with 6 attached rods of different length and PE/PC ratio [24], [54] depending on growth conditions. Assuming an identical core structure, the PBS rods of the *A. caldariorum*-like strain CCALA 981 were almost 40% longer in comparison to PCC 7421 (In Table 3, almost 8 PC+PC trimers per single rod of CCALA 981 and only five PC+PC trimers in PCC 7421). Alternatively, some PBSs without the APC core could occur similarly to the so called Cpc-G2 phycobilisomes recently described for *Synechocystis* sp. [55].

**Table 2:** Relative concentration of particular phycoerythrobilins in phycobilisomes of *Aphanothece caldariorum*-like and *Gloeobacter violaceus* strains calculated from absorption spectra.

	PE/APC	PC/APC	PE/PC
***A. caldariorum*** **-like CCALA 981**	1.71±0.16	1.34±0.21	1.28±0.08
***G. violaceus*** ** PCC 7421**	1.26±0.16	1.06±0.22	1.2±0.11

PE, phycoerythrin; APC, allophycocyanin; PC, phycocyanin. See Methods for precise description of calculation of the values.

**Table 3:** Number of phycoerythrin and phycocyanin trimers in a single phycobilisome rod of *Aphanothece caldariorum*-like and *Gloeobacter violaceus* strains.

	n(PE)	n(PC)
***A. caldariorum*** **-like CCALA 981**	4.5±0.4	3.6±0.6
***G. violaceus*** ** PCC 7421**	3.4±0.4	2.8±0.6

PE, phycoerythrin; PC, phycocyanin. Values were calculated on the basis absorbance changes taking into account a model of the phycobilisome for *G. violaceus* PCC 7421 consisting of the phycobilisome core (16 allophycocyanins trimers) with 6 attached rods with different amount of PE and PC trimers [24].

Our data clearly suggests that the color variability in young cultures (grey/blue-green/violet hues) can be explained by different ratios of individual phycobilins depending on actual growth conditions and physiological state of the cultures (Figure 3A, Figure S3). The versatile phycobilin ratio can also explain the greyish color of young PCC 7421 (Figure 1F), which differs from bright violet color typically described for this strain [6]. As documented by absorption spectra (Figure 3B, C), in older batch cultures the PBSs degraded while carotenoids accumulated. The decrease, and finally the total absence of phycoerytrin (565 nm) and phycocyanin (620 nm) peak, could be seen in the whole cell spectra of older cultures in both CCALA 981 and PCC 7421, where the phycobilins were replaced by an intensively absorbing band in the wavelength range of 400–550 nm, corresponding mainly to carotenoid absorption. This process was reflected by a gradual shift in color from greenish (chlorophyll) to yellowish and orange (carotenoids).

The carotenoid composition of the *A. caldariorum*-like strain CCALA 981 was identical to that of *G. violaceus* PCC7421 as assessed by HPLC analysis. The dominant carotenoids were β-Carotene and (2S,2′S)-oscillol 2,2′-di(α-l-fucoside), while echinenone was found as a minor component. Our results matched those in the report for *G. violaceus* PCC7421 by Tsuchiya and co-workers [16]. Presence of the rarely occurring carotenoid (2S,2′S)-oscillol 2,2′-di(α-l-fucoside) in the studied strains further supported their isolated position in phylogeny of cyanobacteria. Carotenoids belonging to oscillol 2,2-diglycosides were found only in limited number of cyanobacteria and bacteria [56], [57].

### Ultrastructure

**Figure 4:**
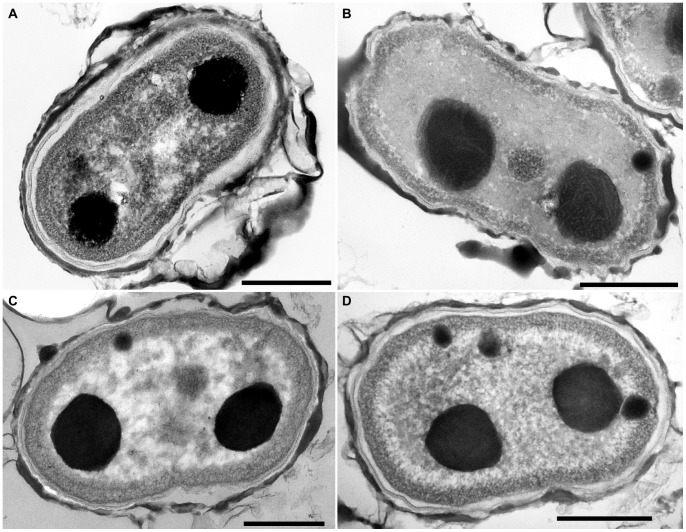
Comparison of cell ultrastructure in *Aphanothece caldariorum*-like strains and ***Gloeobacter violaceus***. Typical cell ultrastructure of *G. violaceus* PCC 9601(A), *G. violaceus* CCALA 979 (B), *A. caldariorum*-like strain CCALA 981(C) and *A. caldariorum*-like strain CCALA 980 (D), respectively. Cells did not contain any thylakoid, the photosynthetic pigments accumulated in an electron-dense layer near the multi-layered cell wall. Cells typically contained two large polyphosphate granules in polar positions. Observed ultrastructure was identical to the reference strain *G. violaceus* PCC 7421 [6]. Scale bars, 500 nm.

Perhaps the most remarkable feature in *Gloeobacter*, that separates it from other cyanobacteria, is the complete absence of thylakoid membranes [6], [58]. Hence, the study of cell ultrastructure by TEM was one of the crucial components of our analysis. Our results unambiguously showed an identical cellular structure in all of the studied strains (Figure 4A–D), exactly matching that of the reference strain PCC 7421 [6]. The multi-layered cell wall was fringed by a band of electron-dense material (photosynthetic pigments). No thylakoid membranes were registered but usually two large polyphosphate granules were present, one at each pole of the cell.

### Phylogeny and Taxonomy

**Figure 5:**
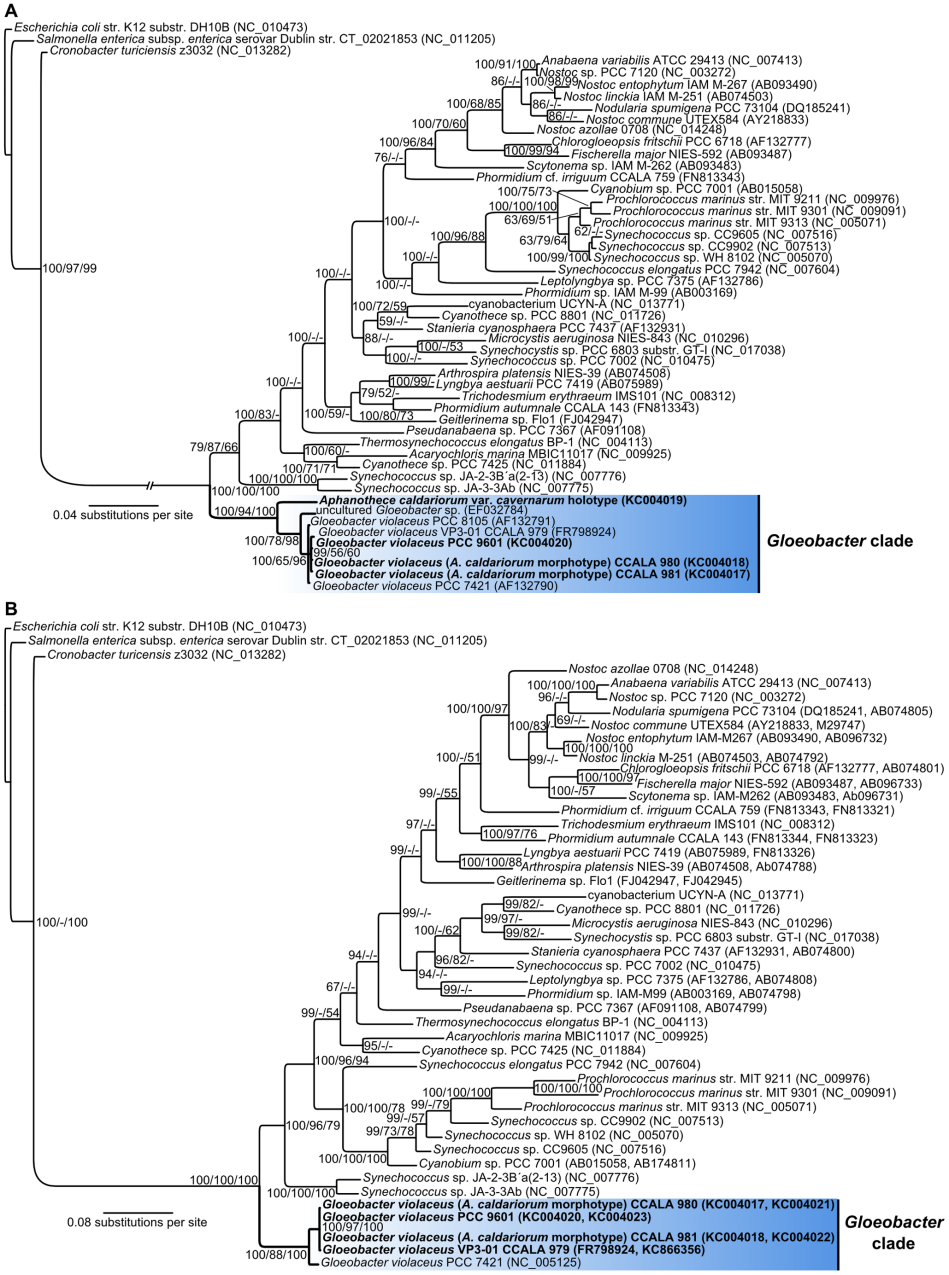
Phylogenetic position of *Gloeobacter violaceus* and *Aphanothece caldariorum*. (A) Phylogenetic tree based on a SSU rRNA gene alignment. (B) Phylogenetic tree based on a concatenated SSU rRNA gene+*rpo*C1 alignment. Sequences generated in this study are printed in bold font. Branch support values (%) are given at nodes in this format: Bayesian inference/maximum likelihood/maximum parsimony. A well supported basal clade of cyanobacteria consisting of *G. violaceus* and *A. caldariorum* is highlighted by blue colour.

The phylogenetic reconstruction based on both, the SSU rRNA gene and a combination of SSU rRNA gene with a protein-coding housekeeping gene like *rpo*C1, provided congruent results regarding the position of *Gloeobacter violaceus* and *Aphanothece caldariorum*-like strains. All studied samples clustered in a single, distinct, fully supported basal clade, which clearly corresponded to the genus *Gloeobacter* (Figure 5). All the cultured strains of *G. violaceus* and *A. caldariorum*-like isolates, including the reference strain PCC 7421, formed an extremely tight cluster, which has to be regarded as the single species - *G. violaceus* (SSU rRNA gene similarity over 99%). This conspicuous taxon is characterized by a specific combination of characters, i.e. phylogenetic position, absence of thylakoids, pigment composition, life cycle, and ecology (life strategy). Given the fact that direct comparison with the type material of *A. caldariorum* var. *caldariorum* by molecular methods is impossible, the decision whether this species is truly identical with *G. violaceus* depends on interpretation of indirect evidence. In our opinion, at least the assignment of *A. caldariorum* to the genus *Gloeobacter* is clear, and the identity of the two species is quite possible. Unfortunately, the name *Gloeobacter violaceus* was never validly published under the rules of Bacteriological Code, and if identity with *A. caldariorum* was assumed, the epithet “*caldariorum*“ (and probably also “*coerulea*“ from *Gloeothece coerulea*) would have priority over “*violaceus*“ under the Botanical Code. Thus, the nomenclatoric status of the species *Gloeobacter violaceus* is rather unclear and has to be amended by a dedicated study. Interestingly, the SSU rRNA gene sequence of the *A. caldariorum* var. *cavernarum* type specimen was slightly different from the rest of *Gloeobacter* sequences. Considering the SSU rRNA gene similarity (∼ 96%), it could be regarded as a separate species of *Gloeobacter*. However, our observations did not reveal any obvious difference (other than DNA sequence) when compared to the material of *A. caldariorum* from the greenhouses in Liberec and Teplice. The original description [32] distinguished between the varieties on the basis of slight differences in cell dimensions and color, characteristics, which were proven in this study to show great variability. In our opinion, a decisive taxonomic conclusion would require analysis of fresh samples, isolated strains of the var. *cavernarum*, and collection of more data. Nevertheless, based on the relatively common occurrence of the *A. caldariorum* morphotype (as mentioned in scientific reports worldwide), it is quite probable that members of the genus *Gloeobacter* are much more common than previously thought. This hypothesis is yet to be tested by careful study of epilithic cyanobacterial communities in future.

While the picture of *Gloeobacter* as an isolated ancient lineage agrees with most published results [4], [12], [13], [59], [60], a recent study [61] proposed a possible relationship to other simple coccoid cyanobacteria. In their report, Courdeau et al. [61] described a phylogenetic cluster with moderate branch support, consisting of *Synechococcus*-like morphotypes related to *Gloeobacter*, which they called 'Gloeobacterales'. It also included the peculiar candidatus *Gloeomargarita lithophora* that forms intracellular calcite precipitates. In a manner similar to *Gloeobacter*, members of this group diverged earlier in evolution than the chloroplast ancestors. In our evolutionary trees, this cluster, represented by the *Synechococcus* strains isolated from the Yellowstone National Park hot springs (C9, Ja-3-3Ab and Ja-2-3B**′**a), branched separately (Figure 5B). Considering the major differences in cell ultrastructure and photosynthetic apparatus, we propose that the order Gloeobacterales should be reserved for the genus *Gloeobacter*, which is primarily defined by the absence of thylakoids [62]. This is further supported by a considerable SSU rRNA gene sequence distance; for comparison, there is approximately 88% similarity between *G. violaceus* PCC 7421 and *Synechococcus* sp. Ja-3-3Ab while the similarity between PCC 7421 and a heterocytous cyanobacterium, *Nostoc* sp. PCC 7120, is 87%. On the other hand, in all known cyanobacteria from these ancient lineages, there is a clear common tendency to colonize wet or submerged, mostly calcite rocks [61]. This evidence reflects a possible first appearance of cyanobacteria in rock-associated, calcifying biofilm habitats, such as stromatolites or travertine spring mats.

### Conclusions

Our results brought new clues to solving a 40 years old puzzle about the true biological identity of *Gloeobacter violaceus*, an important model organism with a great value in several biological disciplines. In the first place, we showed that the genus *Gloeobacter* is a commonly occurring terrestrial cyanobacterium. On the basis of detailed morphological, ultrastructural, biochemical, and phylogenetic comparisons of two available *Gloeobacter* strains, three new independent isolates, and a botanical type specimen, we generated complementary evidence of the identity of *Gloeobacter* with a long known rock-dwelling cyanobacterial morphospecies *Aphanothece caldariorum*. The life strategy of *Gloeobacter/A. caldariorum* is congruent with that of other primitive coccoid cyanobacteria, suggesting a possible origin of their cyanobacterial ancestors in alkaline rock-associated biofilms. In this paper we provided observations of previously unknown genetic variability and phenotypic plasticity, which we expect to be utilized by experimental and evolutionary researchers worldwide.

## Supporting Information

Figure S1 ***A. caldariorum*** **-like morphology: formation of nanocyte-like cells in culture.** The cells of *A. caldariorum* CCALA 981 started rapid successive binary fission shortly after inoculation on fresh media. (A) Cell division into multiple small spherical cells. (B) Nanocyte-like daughter cells forming clusters on solid medium. Formation of nanocyte-like cells was observed in the initial stages of cultivation directly on the agar plate when there was still some contamination by bacteria and fungi. Scale bars, 50 µm.
(PDF)

Figure S2 **Identical morphology of** ***Aphanothece caldariorum-*** **like and** ***Gloeobacter violaceus*** **isolates in culture.** (A) *G. violaceus* PCC 9601; (B) *G. violaceus* CCALA 979; (C) *A. caldariorum*-like strain CCALA 980. Strains PCC 7421 and CCALA 981 are documented in Figure 1. Scale bars, 10 µm.
(PDF)

Figure S3 **Proportion of phycobiliproteins in** ***Aphanothece caldariorum*** **and** ***Gloeobacter violaceus*** **during the culture senescence.** (A) *A. caldariorum* CCALA 981, (B) *G. violaceus* PCC 7421. Both PE and PC were degraded as the culture aged. Thus, the blue-green/violet colour at the beginning of the cultivation was replaced by yellow-orange colour (carotenoids). Interestingly, the PE/PC ratio was increased in old cultures at the end of the cultivation in both strains. This was due to major decrease in PC, as seen from the PC/APC curve. The relatively high PE proportion at the end of cultivation also agreed with the orange colour. PE, phycoerytrin; PC, phycocyanin; APC, allophycocyanin.
(TIF)

Figure S4 **PCR products of SSU rRNA gene region and partial** ***rpo*** **C1 visualized on 1.5% agarose gels.** (A) and (B) SSU rRNA gene region PCR products; (C) and (D) Partial *rpo*C1 gene PCR products; (E) SSU rRNA gene region products amplified from *A. caldariorum* var. *cavernarum* type specimen by direct PCR. Sample names are indicated at loading wells. A standard 100 bp DNA ladder with fragment sizes corresponding to 100, 200, 300, 400, 500, 600, 700, 800, 900, 1000, 1200 and 1517 bp was used in all gels. The samples were stained by GelRed Nucleic Acid Dye (Biotium, Hayward, USA). C, negative control (blank); X, unsuccessful PCR.
(TIF)
