# Supplementary figures and images for: The Primitive Thylakoid-Less Cyanobacterium Gloeobacter Is a Common Rock-Dwelling Organism
Source: PLoS One. 2013 Jun 18;8(6):e66323. doi: 10.1371/journal.pone.0066323 (PMC3688883; doi:10.1371/journal.pone.0066323)

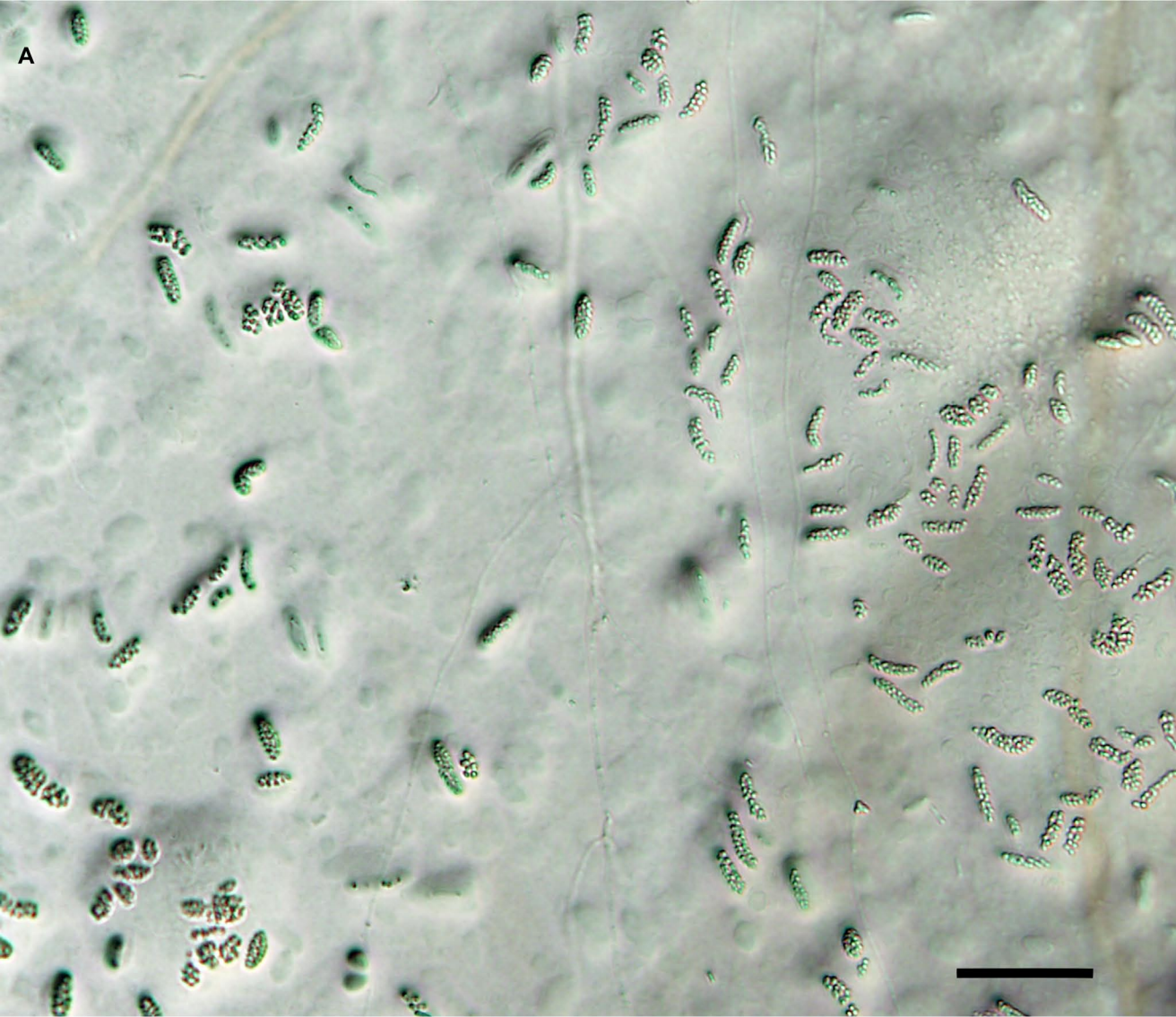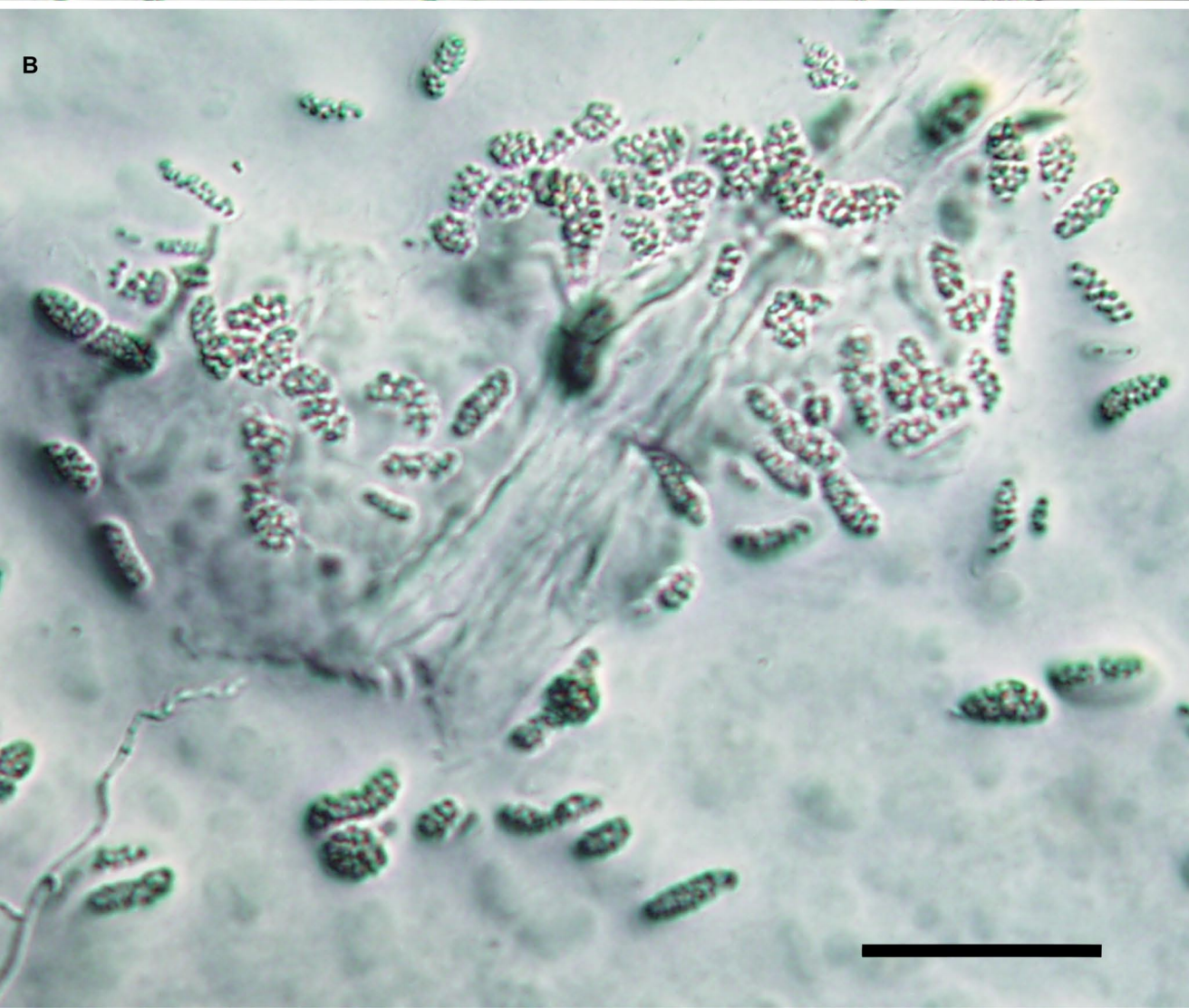

Supplement: Figure S1 — A. caldariorum -like morphology: formation of nanocyte-like cells in culture. The cells of A. caldariorum CCALA 981 started rapid successive binary fission shortly after inoculation on fresh media. (A) Cell division into multiple small spherical cells. (B) Nanocyte-like daughter cells forming clusters on solid medium. Formation of nanocyte-like cells was observed in the initial stages of cultivation directly on the agar plate when there was still some contamination by bacteria and fungi. Scale bars, 50 µm. (PDF) [file pone.0066323.s001.pdf]

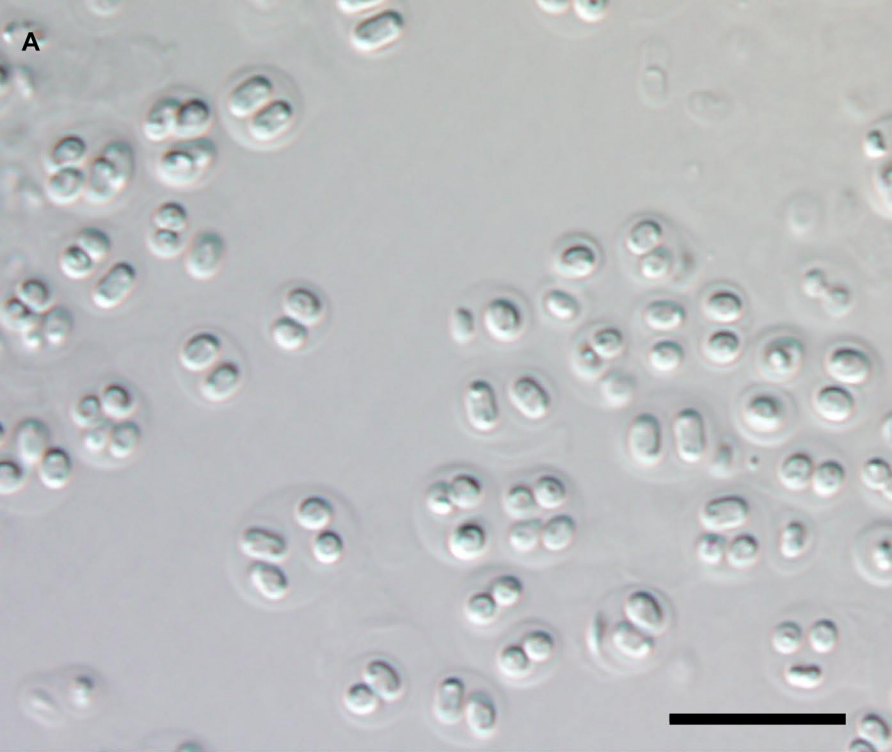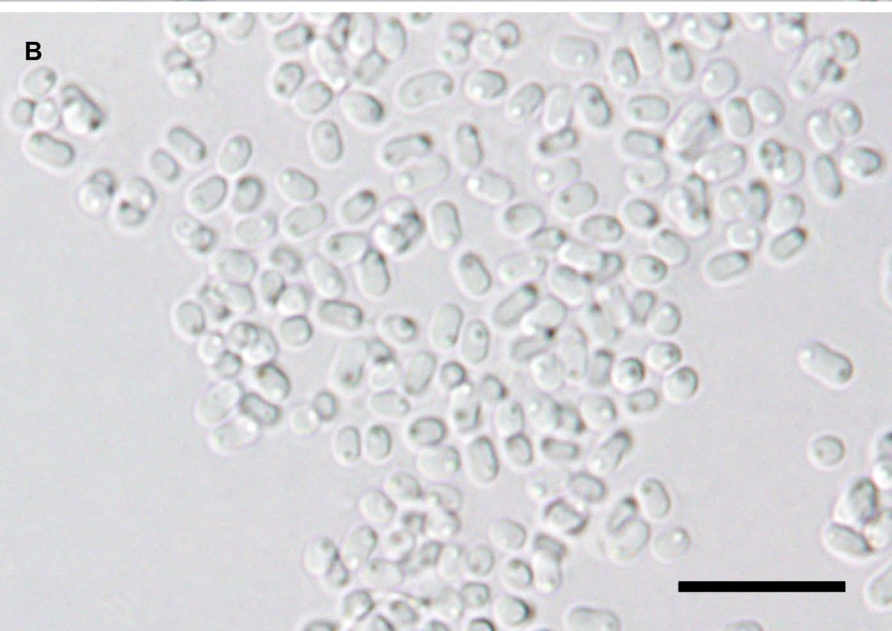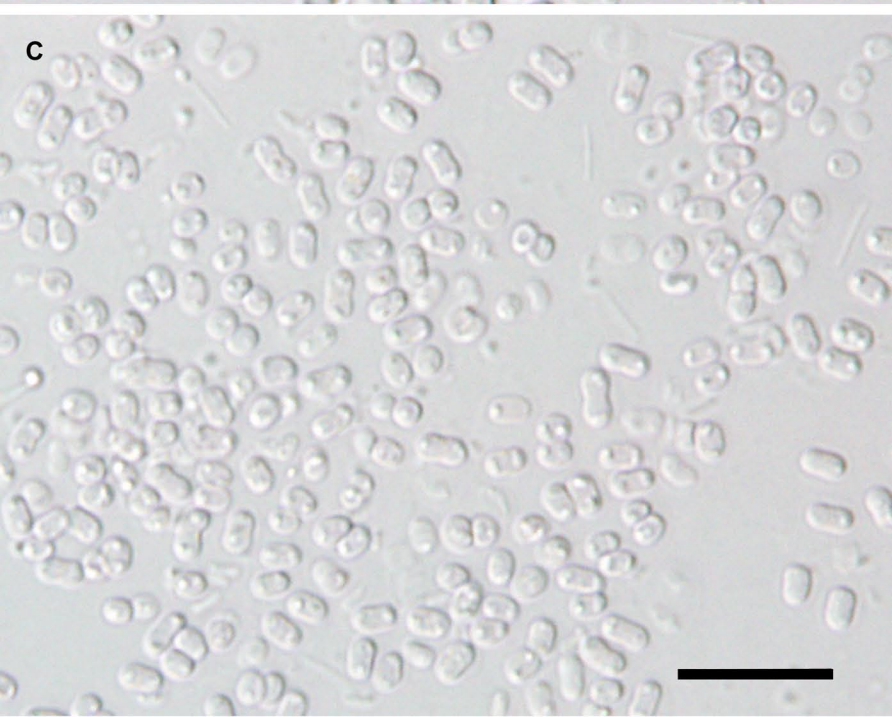

Supplement: Figure S2 — Identical morphology of Aphanothece caldariorum- like and Gloeobacter violaceus isolates in culture. (A) G. violaceus PCC 9601; (B) G. violaceus CCALA 979; (C) A. caldariorum-like strain CCALA 980. Strains PCC 7421 and CCALA 981 are documented in Figure 1. Scale bars, 10 µm. (PDF) [file pone.0066323.s002.pdf]

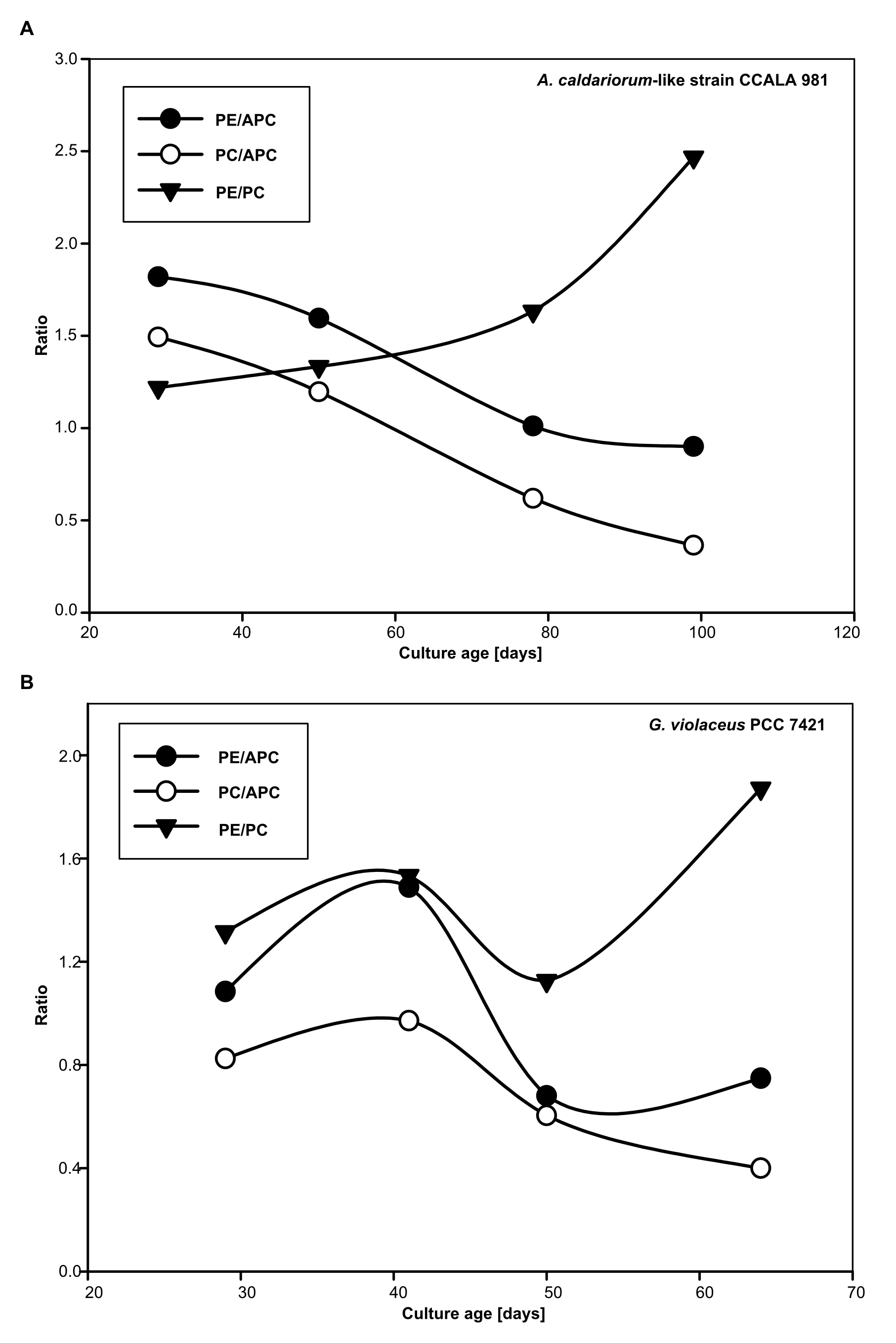

Supplement: Figure S3 — Proportion of phycobiliproteins in Aphanothece caldariorum and Gloeobacter violaceus during the culture senescence. (A) A. caldariorum CCALA 981, (B) G. violaceus PCC 7421. Both PE and PC were degraded as the culture aged. Thus, the blue-green/violet colour at the beginning of the cultivation was replaced by yellow-orange colour (carotenoids). Interestingly, the PE/PC ratio was increased in old cultures at the end of the cultivation in both strains. This was due to major decrease in PC, as seen from the PC/APC curve. The relatively high PE proportion at the end of cultivation also agreed with the orange colour. PE, phycoerytrin; PC, phycocyanin; APC, allophycocyanin. (TIF) [file pone.0066323.s003.tif]

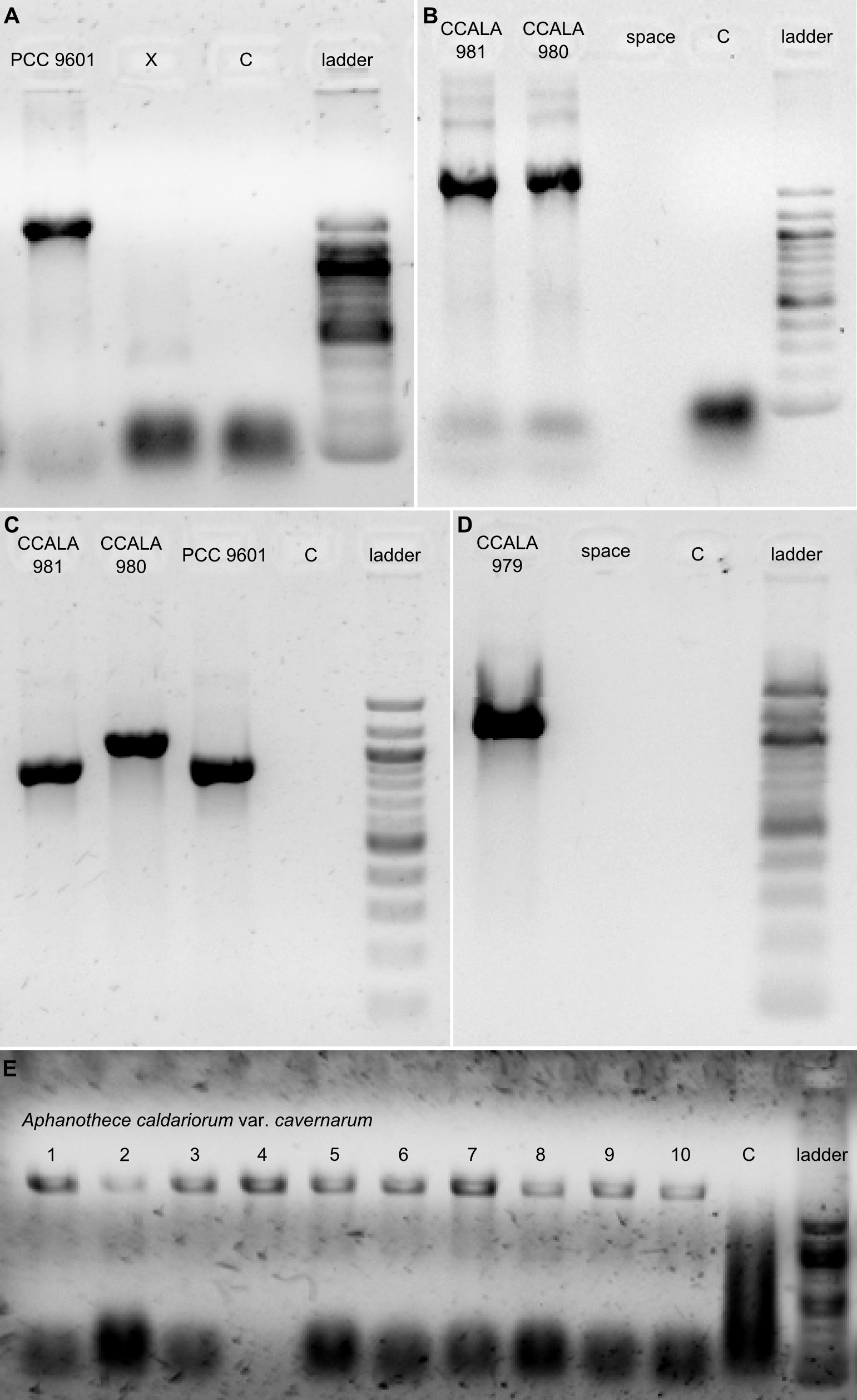

Supplement: Figure S4 — PCR products of SSU rRNA gene region and partial rpo C1 visualized on 1.5% agarose gels. (A) and (B) SSU rRNA gene region PCR products; (C) and (D) Partial rpoC1 gene PCR products; (E) SSU rRNA gene region products amplified from A. caldariorum var. cavernarum type specimen by direct PCR. Sample names are indicated at loading wells. A standard 100 bp DNA ladder with fragment sizes corresponding to 100, 200, 300, 400, 500, 600, 700, 800, 900, 1000, 1200 and 1517 bp was used in all gels. The samples were stained by GelRed Nucleic Acid Dye (Biotium, Hayward, USA). C, negative control (blank); X, unsuccessful PCR. (TIF) [file pone.0066323.s004.tif]
